# Risperidone and Levothyroxine for Managing “Myxedema Madness”

**DOI:** 10.7759/cureus.10152

**Published:** 2020-08-31

**Authors:** Rikinkumar S Patel, Arpit S Heer, Aquila Lesko, Sung W Kim, Muhammad Ishaq

**Affiliations:** 1 Psychiatry, Griffin Memorial Hospital, Norman, USA; 2 Psychiatry and Behavioral Sciences, Jawaharlal Nehru Medical College, Belgaum, IND; 3 Psychiatry, Saint James School of Medicine, Arnos Vale, VCT

**Keywords:** hypothyroidism, psychosis, atypical antipsychotic, myxedema

## Abstract

Hypothyroidism is one of the common comorbidities seen in patients with psychiatric conditions. Sometimes few patients may present with neuropsychiatric symptoms such as cognitive slowing, depression, or psychosis (“myxedema madness”). These patients are managed with antipsychotic medications while admitting laboratory works are processed. It has been found that antipsychotic use is associated with lower free thyroxine levels, so untreated hypothyroid patients may experience worsening of symptoms with antipsychotic use. It is recommended that hypothyroid patients with psychosis be treated for the underlying hypothyroidism with thyroid hormone replacement. In this article, we are presenting a case of a hypothyroid patient presenting to a psychiatric facility for worsening psychosis and persecutory delusions, and medication non-compliance to levothyroxine. We also discuss the management of psychosis in a patient with worsening hypothyroidism with a combination regimen: levothyroxine and risperidone.

## Introduction

Hypothyroidism is one of the most common endocrine disorder, and nearly 0.3% to 0.4% of the population in the western world suffer from overt hypothyroidism. The most common etiology of hypothyroidism is Hashimoto’s disease, in which the immune system attacks the thyroid gland [1]. Other causes include ablation of the thyroid using radioactive iodine, injury or removal of the thyroid during surgery or hypothalamic/pituitary gland dysfunction [2].

It more commonly presents as a wide range of physical symptoms and signs which include cold intolerance, fatigue, constipation, slow heart rate, weight gain, menstrual irregularities, dry skin, hair loss, limb swelling, etc. However, a minority of cases, i.e. 5% to 15% present with neuropsychiatric symptoms including slowing of thought and speech, decreased attentiveness, apathy, depression, agitation, and psychosis “myxedema madness” [2]. 

“Myxedema madness” with cognition problems is seen in hypothyroidism due to the accumulation of glycosaminoglycans in body tissues, and added to this a higher mean glycosaminoglycan concentration is seen in schizophrenic patients [3,4]. There is a high concentration of thyroid triiodothyronine (T3) receptors in the amygdala and hippocampus that have a direct influence on neural activity [5]. Where the amygdala is crucial in regulating emotional response, the hippocampus plays a major role in learning and memory development [6,7]. Hypothyroidism is associated with an increase in cerebral dopamine levels and tyrosine hydroxylase activity, and similarly increased subcortical dopamine is seen in schizophrenic patients with predominantly positive symptoms [8,9].

We present a case of a middle-aged female with increased psychosis after stopping her thyroid hormone medication (levothyroxine). Next, we discuss the management of psychosis in a patient with worsening hypothyroidism.

## Case presentation

Our patient is a 52-year-old female with a past psychiatric history of schizophrenia and post-traumatic stress disorder (PTSD) who was transferred to Griffin Memorial Hospital (GMH) on court commitment status for further inpatient treatment of worsening psychosis and suicidal ideations. During the initial evaluation, the patient endorsed religious delusions with a feeling of spiritual awakening and the ability to communicate with God. She endorsed bizarre delusions and auditory hallucinations about hearing radio frequencies. The patient has a medical history of graves’ disease, and radioactive iodine thyroid ablation in 1993. Prior to inpatient hospitalization, the patient was non-compliant with levothyroxine for three months due to jealous delusions and persecutory delusions about her husband trying to poison her medications.

The patient was resumed on duloxetine 30mg daily for depression, valproate 500mg twice daily (BID) for mood stabilization and irritability, and risperidone 2mg BID for psychosis. Laboratory reports with complete blood count, comprehensive metabolic profile, lipid panel, hemoglobin a1c, and urine drug screen revealed no acute abnormalities, and thyroid function test was pending.

On day three, the patient continued to have persecutory and jealous delusions and is considering divorce after 23 years of marriage, but the patient could not elaborate more on details about the divorce. The patient’s psychosis was worsening; the patient had the ability to receive special messages from God, and the ability to read people’s energy and mind as she was gifted with “psyche and high-functioning autism”. Risperidone was increased to 3mg BID for psychosis, and valproate was tapered down to 500mg at night time (qHS) as the patient had a stable mood and she complained of daytime drowsiness. On day seven, the patient reported increased fatigue and memory problems. Mini-mental status examination (MMSE) was conducted and she scored a 24 out of 30, compared to the initial MMSE of 27. Physical examination showed cold extremities, thinning hair, and dry skin/scalp. Her subsequent lab showed thyroid-stimulating hormone (TSH) level 50.8 milliunits per liter (mU/l) (normal reference range: 0.5-5.0 mU/l), thyroxine (T4) level <1.2 micrograms per deciliter (μg/dl) (normal reference range: 5.0-12.0μg/dl), cholesterol level 357mg/dl (normal reference range: less than 170mg/dl) and triglycerides level 292mg/dl (normal reference range: less than 150mg/dl). Patient was started on levothyroxine 25μg in the morning (qAM) for hypothyroidism with a plan to titrate by an increment of 25μg every three days up to a total dose of 150μg qAM, and was also started on atorvastatin 20mg qAM for hyperlipidemia.

On day 15, the patient’s paranoia, and religious delusions were resolved, but she continued to endorse fixed jealous delusion but were less compared to the past week. The patient denied persecutory delusions and stated that she felt safe on the unit. Patient complained of no changes in cognitive slowing but improved with cold intolerance and energy levels. Subsequent laboratory results showed TSH level 40.0 mU/l (normal reference range: 0.5-5.0 mU/l), T4 level 3.8 μg/dl (normal reference range: 5.0-12.0μg/dl), free T3 level <1.0 nanograms per deciliter (ng/dl) (normal reference range: 0.2-0.5 ng/dl), and free T4 level 0.57 ng/dl (normal reference range: 0.8-2.8 ng/dl). The patient was started on haloperidol 5mg BID for psychosis and to augment the antipsychotic effect with risperidone 3mg BID.

On day 20, the patient states that current psychotropic regimen helped her to keep her thoughts “clear”, and denies having any jealous and persecutory delusions, and feels safe going home with her husband. Valproate was discontinued and haloperidol was tapered down to 5mg qAM and then discontinued. On day 22, the patient reported that her mood and energy had improved, and denied paranoia, delusions, and psychosis. The patient denied suicidal or homicidal ideations with no intent/plan. Patient had a linear and goal-directed thought process, with improved insight and judgment, and agreed to be compliant with her psychotropic regimen by following up at outpatient psychiatry, and was discharged to her husband’s home.

## Discussion

When a patient with acute psychosis presents to a psychiatric hospital, it is common to start a patient with an antipsychotic medication while waiting for blood lab results to come back. The use of antipsychotics was significantly associated with lower free thyroxine (fT4) level (P = 0.001), but not with the TSH levels. They also found significant associations between lower fT4 level and current use of quetiapine (P = 0.005) and olanzapine (P = 0.018), but again no significant associations were found with TSH level [10]. Measuring the TSH level is indicated when hypothyroidism is suspected and is confirmed if TSH is high; and T4 levels are used to distinguish between primary hypothyroidism (low T4) or secondary hypothyroidism. The treatment for hypothyroidism is thyroid hormone replacement with synthetic T4 or a T3/T4 combination [1,5]. For those with symptoms of psychosis, it is recommended to treat the underlying hypothyroidism first. The psychosis should begin abating within a week of thyroid hormone treatment, but a delay in hypothyroid treatment may cause a failure in remission of psychotic symptoms [11]. Additionally, adding an antipsychotic with thyroid hormone replacement may fasten the remission of psychosis [12].

There are few case reports on the treatment course of psychosis secondary to hypothyroidism, and to the best of our knowledge, there are none in which antipsychotic treatment was initiated weeks before thyroid hormone replacement. Hynicka published a case report in which a female patient with a past medical history of severe hypothyroidism presented with auditory hallucinations and delusions and a TSH level of 60.29 mU/l (normal reference range: 0.5-5.0 mU/l). The patient was started on levothyroxine 50μg intravenously (IV) but lacked improvement in psychosis for six days, and then it was decided to start haloperidol 0.5mg daily. On day ten, haloperidol was increased to 1mg daily, and the patient improved with psychosis [2]. Maksim et al. reported a 65-year-old female patient admitted for capgras syndrome, catatonia, and hallucinations with a TSH level of 100.34 mU/l (normal reference range: 0.5-5.0 mU/l). The patient was started on levothyroxine 50μg and titrated to 100μg with little improvement in psychosis in eight weeks even though euthyroid status was attained. Olanzapine 10mg daily was added and the patient experienced remission of psychosis within three weeks [3]. Juneja and Nance discussed a 34-year-old female patient with a past surgical history of total thyroidectomy who presented with grandiose, persecutory, and bizarre delusions along with tactile and visual hallucinations [13]. With a TSH of >100 mU/l (normal reference range: 0.5-5.0 mU/l), the patient was started on initial high doses of T4 with oral T3 and quetiapine 800mg daily. She achieved a euthyroid state in two weeks but only saw improvement in psychiatric symptoms at one month.

In this case report, our patient demonstrated improvement in jealous, paranoid and persecutory delusions and auditory hallucinations on risperidone 3mg BID (for three weeks) and levothyroxine 150μg daily (for two weeks) even though a euthyroid state was not attained before discharge. Risperidone is a benzisoxazole derivative with an antipsychotic property that antagonizes serotonin effects via the serotonin 5-ht2 receptors and, to a lesser extent, competes with dopamine at the limbic dopamine d2 receptors. It also has a low to moderate efficacy affinity for histaminic h1 and serotonin (5ht1a, 5-ht1c, and 5-ht1d) receptors with weak affinity for dopamine d1 receptor and haloperidol-sensitive stigma sites. The decreased serotonergic and dopaminergic pathway activity is responsible for the decrease in both psychotic and mood symptoms [14].

## Conclusions

Although uncommon, hypothyroidism can present as changes in mental status and cognition, a condition that is termed as "myxedema madness". The physical symptoms usually resolve faster whereas the psychotic symptoms may persist even when euthyroid status is attained. Using antipsychotics prematurely in such cases can have a further negative impact on the free thyroxine levels. Therefore, if the laboratory results indicate hypothyroidism, the best next step is to start the patient on thyroid hormone replacement (levothyroxine). Risperidone can be the preferred antipsychotic in combination with levothyroxine to fasten the return to a psychiatric baseline.
